# ﻿Morphology and multi-gene phylogeny reveal three new species of *Clonostachys* and two combinations of *Sesquicillium* (Bionectriaceae, Hypocreales) from Xizang, China

**DOI:** 10.3897/mycokeys.115.139757

**Published:** 2025-03-11

**Authors:** Shucheng He, Vinodhini Thiyagaraja, Chitrabhanu S. Bhunjun, Putarak Chomnunti, Lakmali S. Dissanayake, Ruvishika S. Jayawardena, Hongde Yang, Yun Wei Zhao, Fatimah Al-Otibi, Qi Zhao, Kevin D. Hyde

**Affiliations:** 1 State Key Laboratory of Phytochemistry and Natural Medicines, Kunming Institute of Botany, Chinese Academy of Sciences, Kunming, Yunnan 650201, China Mae Fah Luang University Chiang Rai Thailand; 2 Center of Excellence in Fungal Research, Mae Fah Luang University, Chiang Rai 57100, Thailand Chinese Academy of Sciences Yunnan China; 3 Center for Mountain Futures, Kunming Institute of Botany, Chinese Academy of Sciences, Honghe County 654400, China Chinese Academy of Sciences Honghe County China; 4 Department of Economic Plants and Biotechnology, Yunnan Key Laboratory for Wild Plant Resources, Kunming Institute of Botany, Chinese Academy of Sciences, Kunming 650201, Yunnan, China Mae Fah Luang University Chiang Rai China; 5 State Key Laboratory for Conservation and Utilization of Bio-Resources in Yunnan, Yunnan University, Kunming, Yunnan, 650091, China Yunnan University Yunnan China; 6 Department of Botany and Microbiology, College of Science, King Saud University, P.O. Box 22452, Riyadh 11495, Saudi Arabia King Saud University Riyadh Saudi Arabia

**Keywords:** Asexual morph, endophytes, Hyphomycetes, new taxa, taxonomy

## Abstract

*Clonostachys* and *Sesquicillium* are genera in Bionectriaceae, and known in sexual perithecial ascomata and hyphomycetous asexual morphs. In their asexual morph, both genera share similar morphology in conidiophores and conidiogenous cell characteristics but differ in the development of conidiophores. The members of *Clonostachys* are distributed worldwide with the majority occurring in the tropics and the species are commonly reported as soil-borne fungi but also reported as endophytes, epiphytes, and saprotrophs. During a microfungi survey in Xizang, China, six collections of fresh and healthy *Ageratinaadenophora* and *Houttuyniacordata* leaves were obtained. The taxonomy of these collections was investigated through a combination of morphological analysis and multigene phylogenetic analysis using Maximum likelihood and Bayesian inference. The newly generated sequences were clustered within *Clonostachys* and *Sesquicillium*, showing hyphomycetes asexual morph. The results revealed three new *Clonostachys* species viz, *Clonostachyslinzhiensis*, *C.motuoensis*, and *C.yadongensis*. This research sheds light on the overlooked fungal diversity in Xizang, China, expanding the known fungal biodiversity in the region. Additionally, two new combinations, *Sesquicilliumaquaticum* and *S.shanghaiense* for *C.aquatica* and *C.shanghaiensis*, and one synonymy, *C.viticola* for *C.swietenia* are established, respectively.

## ﻿Introduction

*Clonostachys* (Bionectriaceae, Hypocreales) was established by Corda (1839). The genus was typified by *C.araucaria* (Corda 1839), which was later synonymized under *C.rosea* (Rossman et al. 2013). The genus was considered as the asexual morph of *Bionectria* and both genera were also considered as conspecific in several studies (Luo and Zhuang 2007, 2010; Dong et al. 2023). *Bionectria* was described by Spegazzini (1918). Based on the One Fungus = One Name (1F = 1N) concept, mycologists propose the protection of the older asexual morph-typified name *Clonostachys* for this genus (Rossman et al. 2013; Dong et al. 2023). Members of *Clonostachys* occur as endophytes, entomopathogens, epiphytes, plant pathogens, soil-borne fungi, and saprotrophs, typically found on herbicolous, corticolous, lichenicolous, fungicolous, coprophilous habitats as well as on nematodes and insects (Mazen et al. 2022; Dong et al. 2023; Wang et al. 2023; Zhao et al. 2023). They are distributed globally and commonly occur in tropical regions (Schroers 2001). The sexual morph is characterized by ascomata that do not change colour in 3% Potassium Hydroxide (KOH) or 100% Lactic Acid (LA) (Luo and Zhuang 2007, 2010), perithecial or cleistothecial ascomata that are superficial on the substrate or embedded in the stroma. Ascomata are solitary or densely aggregated, subglobose to pyriform; clavate or cylindrical, sessile or short pedicellate asci, smooth or striated, aseptate to multi-septate, globose, fusiform, ellipsoid or broadly ellipsoid ascospores (Hyde et al. 2020a). The asexual members are characterized by penicillate, sporodochial and dimorphic conidiophores (primary and secondary conidiophores) with phialidic conidiogenous cells, hyaline, smooth, broadly ellipsoidal conidia with ends that are broadly rounded (Bao et al. 2023; Chen et al. 2023; Dong et al. 2023; He et al. 2023; Liu et al. 2023; Perera et al. 2023). Primary conidiophores are mononematous, either verticillium-like or narrowly penicillate, whereas the secondary conidiophores produce imbricate conidial chains that can collapse to slimy masses, particularly on sporodochia (Zhao et al. 2023).

Morphology-based identification of *Clonostachys* is challenging (Schroers et al. 1999; Abreu et al. 2014) and many species were previously placed in various genera such as *Acrostalagmus*, *Clonostachyopsis*, *Dendrodochium*, *Gliocladium*, *Gliocladochium*, *Myrothecium*, *Sesquicillium*, *Spicaria*, *Verticilliodochium*, or *Verticillium* (Schroers 2001). Rossman et al. (2001) first conducted the initial molecular investigation of *Clonostachys*/*Bionectria*, employing large subunit rDNA sequences, and proposed the monophyletic status. Subsequently, DNA sequences from multi-genes including ITS, 28S, *rpb*1, *rpb*2, and *tef*1 have been extensively employed to address the taxonomy of *Clonostachys* (Bao et al. 2023; Chen et al. 2023; Perera et al. 2023; Zhao et al. 2023). Wijayawardene et al. (2022) accepted 78 species under *Clonostachys*, while this was 50 species in Hyde et al. (2024). Zhao et al. (2023) investigated the species diversity within a collection of 420 strains of *Clonostachys* from the culture collection and personal collections at the Westerdijk Fungal Biodiversity Institute in Utrecht, the Netherlands, and identified 19 species based on phylogenetic and morphological analyses. In China, 19 *Clonostachys* species have been reported from different hosts and substrates (Bao et al. 2023; Dong et al. 2023; Perera et al. 2023; Piombo et al. 2023; Wang et al. 2023).

During the microfungi survey in China (He et al. 2024a, b, c; Thiyagaraja et al. 2024), we investigated several isolates from the leaves of *Ageratinaadenophora* and *Houttuyniacordata* from Xizang, China. Multigene phylogenetic analyses combining 28S, *tef*1, *rpb*2, ITS, and *tub*2 sequences, along with morphological analyses, support the establishment of three new species: *Clonostachyslinzhiensis*, *C.motuoensis* and *C.yadongensis*. The introduction of these new species follows the protocols outlined in Chethana et al. (2021) and Maharachchikumbura et al. (2021). The new species are established based on detailed morphological characterization, and illustrations, along with multigene analyses of maximum likelihood (ML) and Bayesian inference (BI). In addition, through phylogenetic analysis of *Clonostachys*, we suggest that *C.aquatica*, *C.shanghaiensis*, and *C.swieteniae* be synonymous with *Sesquicilliumaquaticum*, *S.shanghaiense*, and *C.viticola*, respectively.

## ﻿Materials and methods

### ﻿Sample collection, isolation, and morphological characterization

Fresh and healthy leaves of *Ageratinaadenophora* and *Houttuyniacordata* were collected from Medog County, Linzhi City, Xizang Autonomous Region, China from October 2021 to July 2023, and information on collection was recorded according to the Rathnayaka et al. (2024). The healthy part of the leaves was initially cleaned and cut into small pieces (5 × 5 mm). The leaf fragments were briefly soaked in a 75% ethanol solution for 30 s, followed by a 2.5% sodium hypochlorite solution for the same duration (Bhunjun et al. 2021). Afterward, they were washed thrice with sterile distilled water for 30 s. Once sterilized, the tissue fragments were allowed to air-dry on sterile filter paper and then transferred to potato dextrose agar (PDA) (Senanayake et al. 2020). The PDA plates were cultured at 25 °C for 2–5 days. Single hyphae were carefully selected from the periphery of the growing colonies and inoculated onto new PDA plates. Following 1–2 weeks of purification, a pure culture was obtained. Sporulation was induced on water agar (WA) medium. The mycelia were mounted on a slide in water using a sterile needle. A NIKON ECLIPSE Ni-U compound microscope was used to examine conidiophores and conidia of a small mass of mycelia. Micro-morphological images were captured with a DS-Ri2 camera attached to the compound microscope. The photoplates used for the figure were processed with Adobe Photoshop. The pure cultures were deposited in the Kunming Institute of Botany, the Chinese Academy of Sciences (KUNCC), Kunming, China. Specimens were deposited in the Herbarium of Cryptogams, Kunming Institute of Botany, Academia Sinica (KUN-HKAS), Kunming, China. Facesoffungi and Index Fungorum numbers were registered following the protocols outlined in Jayasiri et al. (2015) and Index Fungorum, respectively.

### ﻿DNA extraction, PCR amplification and sequencing

The mycelia growing on a PDA plate were used to extract DNA using the Trilief^TM^ Plant Genomic DNA Kit (Tsingke Biological Technology Co., Ltd in Beijing, China), following the manufacturer’s instructions. The primer pairs ITS5/ITS4 (White et al. 1990), LR0R/LR5 (Vilgalys and Hester 1990), T1/T22 (Research & Service 1997), EF1-983F/EF1-2218R (Carbone and Kohn 1999), and fRPB2-5F/fRPB2-7cR (Liu et al. 1999) were used for amplification of the internal transcribed spacer region ITS1-5.8S-ITS2 (ITS), large subunit rDNA (28S), beta-tubulin (*tub*2), translation elongation factor 1-α (*tef*1) gene and RNA polymerase II second-largest subunit (*rpb*2), respectively. The PCR was performed in a 25 μL reaction volume, comprising 21 μL PCR Mix (2 × Rapid Taq Master Mix, Vazyme Biotech Co., Ltd., Nanjing, China), 1 μL of each primer, 2 μL of DNA template. For PCR amplification conditions see Table 1. The PCR products were visualized using agarose gel electrophoresis, and those with the targeted bands were sent to Sangon Biotech Co. Ltd., Kunming, China, for sequencing. The newly generated sequences were submitted to GenBank to obtain accession numbers.

**Table 1.** Loci, primers, and PCR amplification conditions used in this study.

Locus	Primers	PCR amplification conditions	Reference
ITS	ITS5/ITS4	95 °C: 5 min, (95 °C: 15s, 55 °C: 15s, 72 °C: 15s) × 40 cycles	White et al. (1990); Vilgalys and Hester (1990)
28S	LR0R/LR5		
*tef*1	EF1-983F/EF1-2218R	95 °C: 5 min, (95 °C: 45s, 52 °C: 45s, 72 °C: 70s) × 35 cycles	Carbone and Kohn (1999)
*tub*2	T1/T22	95 °C: 5 min, (95 °C: 45s, 50 °C: 45s, 72 °C: 90s) × 35 cycles	Research and Service (1997)
*rpb*2	fRPB2-5F/fRPB2-7cR	95 °C: 5 min, (95 °C: 45s, 55 °C: 120s, 72 °C: 50s) × 35 cycles	Liu et al. (1999)

### ﻿Sequence alignment and phylogenetic analyses

The sequences were assembled using Sequencing Project Management (SeqMan) software (Clewley 1995). The assembled sequences were compared with the data in GenBank to determine their close relatives. The results indicate that our specimens were closely related to species of *Clonostachys*. Reference sequences for *Clonostachys* were obtained following recent studies (Bao et al. 2023; Liu et al. 2023; Perera et al. 2023; Piombo et al. 2023; Wang et al. 2023; Zhang et al. 2023; Zhao et al. 2023) (Table 2). Each gene matrix was separately aligned using MAFFT v. 6.8 (Katoh et al. 2018). The aligned datasets were manually edited using BioEdit v. 7.0.9 (Hall 1999) and then combined using SequenceMatrix v1.7.8 (Vaidya et al. 2011). The combined alignment was utilized for ML and BI analyses.

**Table 2.** Names, voucher numbers, and corresponding GenBank accession numbers of the taxa used in the phylogenetic analyses in this study.

Taxa	Voucher no.	GenBank accession numbers					Reference
		ITS	28S	*tub*2	*tef*1	*rpb*2	
***Clonostachysagrawalii***	**CBS 533.81**	**AF358241**	N/A	**AF358187**	N/A	N/A	Schroers (2001)
***C.ambigua***	**PAD S00003**	**MT554898**	N/A	**N/A**	N/A	N/A	Forin et al. (2020)
***C.apocyni***	**CBS 130.87**	**AF210688**	N/A	**AF358168**	N/A	N/A	Schroers (2001)
***C.aranearum***	**QLS 0625**	**NR_164542**	N/A	**KU212400**	N/A	N/A	Chen et al. (2016)
***C.artemisiae***	**MHZU 23-0116**	**OR365451**	N/A	**OR700206**	N/A	N/A	Dong et al. (2023)
*C.aurantiaca*	CBS:124757	OQ910531	OQ910890	N/A	OQ944545	OQ927609	Zhao et al. (2023)
*C.aureofilvella*	CBS 195.93	AF358226	N/A	AF358181	N/A	N/A	Schroers (2001)
***C.australiana***	**CBS:102421**	**OQ910540**	**OQ910899**	**OQ982584**	**OQ944554**	**OQ927618**	Zhao et al. (2023)
***C.bambusae***	**CBS:139411**	**OQ910542**	**OQ910901**	**OQ982586**	**OQ944556**	**OQ927620**	Zhao et al. (2023)
***C.buxicola***	**CBS:102419**	**OQ910544**	**OQ910903**	**OQ982588**	**OQ944558**	**OQ927622**	Zhao et al. (2023)
***C.byssicola***	**CBS 364.78**	**MH861151**	**MH872912**	**AF358153**	N/A	N/A	Vu et al. (2019)
***C.capitata***	**CBS 218.93**	**AF358240**	**MH874054**	**AF358188**	N/A	N/A	Schroers (2001)
***C.catenulata***	**CBS 154.27**	**NR_165993**	**NG_063969**	N/A	**OQ944810**	**OQ927866**	Zhao et al. (2023)
***C.chlorina***	**CBS 287.90**	**NR_137651**	**MH873895**	**OQ982590**	**OQ944560**	**OQ927624**	Schroers (2001)
***C.chloroleuca***	**CBS:141588**	**OQ910549**	**OQ910908**	N/A	**OQ944563**	**OQ927627**	Zhao et al. (2023)
***C.chongqingensis***	**HMAS 290894**	**OP205475**	N/A	**OP205324**	N/A	N/A	Zeng and Zhuang (2022)
*C.compactiuscula*	CBS:123759	OQ910563	OQ910922	OQ982603	OQ944576	OQ927640	Zhao et al. (2023)
***C.compactiuscula***	**CBS 913.97**	**AF358245**	N/A	**AF358194**	N/A	N/A	Schroers (2001)
***C.cylindrica***	**CBS:101113**	**OQ910569**	**OQ910928**	N/A	**OQ944582**	**OQ927646**	Zhao et al. (2023)
***C.divergens***	**CBS 967.73**	**NR_137532**	**OQ910934**	**AF358191**	**OQ944587**	N/A	Schroers (2001)
***C.ellipsoidea***	**CBS 175.76**	**OQ910580**	**OQ910939**	**OQ982617**	**OQ944592**	**OQ927655**	Zhao et al. (2023)
***C.epichloe***	**CBS 101037**	**AF210675**	**OQ910940**	**AF358209**	**OQ944593**	**OQ927656**	Schroers (2001)
***C.eriocamporesiana***	**MFLU 18-2713**	**MN699132**	**N/A**	**MN699965**	**MN699964**	N/A	Hyde et al. (2020b)
***C.eriocamporesii***	**MFLU 19-0486**	**MN699133**	**NG_068919**	**OQ982619**	N/A	N/A	Hyde et al. (2020b)
*C.farinosa*	CBS 914.97	AF358252	N/A	AF358151	N/A	N/A	Schroers (2001)
***C.flava***	**CBS 915.97**	**OQ910619**	**OQ910978**	**OQ982654**	**OQ944631**	**OQ927690**	Zhao et al. (2023)
***C.fujianensis***	**CBS:127474**	**OQ910620**	**OQ910979**	**OQ982655**	**OQ944632**	**OQ927691**	Zhao et al. (2023)
***C.fusca***	**CBS 207.93**	**OQ910622**	**OQ910981**	**OQ982657**	**OQ944634**	**OQ927693**	Zhao et al. (2023)
***C.garysamuelsii***	**CBS:123964**	**OQ910624**	**OQ910983**	**OQ982658**	**OQ944636**	**OQ927695**	Zhao et al. (2023)
***C.grammicospora***	**CBS 209.93**	**NR_137650**	**NG_064165**	**AF358206**	**OQ944637**	N/A	Forin et al. (2020)
*C.grammicosporopsis*	CBS 102834	AF358256	OQ910985	OQ982660	OQ944638	OQ927697	Vu et al. (2019)
***C.granuligera***	**PAD S00011**	**MT554904**	N/A	N/A	N/A	N/A	Forin et al. (2020)
***C.hongkongensis***	**CBS:115291**	**OQ910630**	**OQ910989**	**OQ982663**	**OQ944642**	**OQ927700**	Zhao et al. (2023)
*C.impariphialis*	HMAS 275560	KX096609	KX096606	N/A	N/A	N/A	Zeng and Zhuang (2022)
***C.indica***	**RKV2015**	**KT291441**	N/A	N/A	N/A	N/A	Prasher and Chauhan (2017)
***C.intermedia***	**CBS 508.82**	**NR_137652**	**OQ910991**	**AF358205**	**OQ944644**	N/A	Schroers (2001)
***C.kowhai***	**CBS 461.95**	**NR_154748**	**OQ910992**	**AF358170**	**OQ944645**	**OQ927702**	Schroers (2001)
***C.krabiensis***	**MFLU 16-0254**	**NR168189**	**MH376707**	N/A	N/A	N/A	Tibpromma et al. (2018)
*C.krabiensis*	CBS 192.96	OQ910634	OQ910993	OQ982666	OQ944646	OQ927703	Zhao et al. (2023)
***C.kunmingensis***	**YFCC: 898**	**MW199069**	**MW199058**	**MW201676**	**MW295969**	N/A	Wang et al. (2023)
***C.leptoderma***	**HMAS 255834**	**OP205474**	N/A	**OP205323**	N/A	N/A	Zeng and Zhuang (2022)
*C.leucaenae*	MFLU 20-0008	ON230050	ON230058	N/A	N/A	N/A	Perera et al. (2023)
*C.levigata*	CBS 948.97	AF210680	N/A	AF358196	N/A	N/A	Schroers (2001)
***C.linzhiensis***	**HKAS 133179**	**PQ522504**	**PQ634391**	**PQ650459**	**PQ650477**	N/A	present study
*C.linzhiensis*	HKAS 133180	PQ522505	PQ634392	PQ650460	PQ650478	N/A	present study
***C.longiphialidica***	**CBS 112.87**	**OQ910643**	**OQ911002**	N/A	**OQ944655**	**OQ927712**	Zhao et al. (2023)
***C.lucifer***	**CBS 100008**	**AF210683**	**OQ911003**	**AF358208**	**OQ944656**	**OQ927713**	Schroers (2001)
***C.miodochialis***	**CBS 997.69**	**NR_137649**	**NG_064076**	**AF358210**	**OQ944658**	**OQ927715**	Schroers (2001)
*C.moreaui*	CBS:127881	OQ910647	OQ911006	OQ982678	OQ944659	OQ927716	Zhao et al. (2023)
***C.motuoensis***	**HKAS 133181**	**PQ522506**	**PQ634393**	**PQ650461**	**PQ650479**	N/A	present study
*C.motuoensis*	HKAS 133182	PQ522507	PQ634394	PQ650462	PQ650480	N/A	present study
***C.oblongispora***	**CBS 100285**	**AF358248**	**OQ911007**	**AF358169**	**OQ944660**	**OQ927717**	Schroers (2001)
*C.obovatispora*	CBS:118752	OQ910649	OQ911008	OQ982680	OQ944661	OQ927718	Zhao et al. (2023)
***C.oligospora***	**HMAS 290895**	**OP205473**	N/A	**OP205322**	N/A	N/A	Zeng and Zhuang (2022)
***C.pallens***	**PAD S00004**	**MT554899**	N/A	N/A	N/A	N/A	Forin et al. (2020)
***C.palmae***	**CBS 119.87**	**OQ910650**	**OQ911009**	**OQ982681**	**OQ944662**	**OQ927719**	Zhao et al. (2023)
***C.parasporodochialis***	**CBS 192.93**	**OQ910651**	**OQ911010**	**OQ982682**	**OQ944663**	**OQ927720**	Zhao et al. (2023)
***C.penicillata***	**CBS 729.87**	**OQ910654**	**OQ911013**	**OQ982685**	**OQ944666**	**OQ927722**	Zhao et al. (2023)
*C.pilosella*	CLLG19028	N/A	NG_153902	N/A	N/A	N/A	Lechat et al. (2020)
***C.pityrodes***	**CBS 102033**	**AF210672**	**OQ911014**	**AF358212**	N/A	**OQ927723**	Schroers (2001)
***C.pnagiana***	**CLLG19041**	N/A	**NG_153903**	N/A	N/A	N/A	Lechat et al. (2020)
***C.pseudochroleuca***	**CBS 192.94**	**AF358238**	N/A	**AF358171**	N/A	N/A	Schroers (2001)
*C.pseudostriata*	CBS 309.96	OQ910673	OQ911032	OQ982704	OQ944685	OQ927741	Zhao et al. (2023)
*C.pseudostriatopsis*	h116	N/A	N/A	AB237465	N/A	N/A	Hirooka and Kobayashi (2007)
*C.ralfsii*	CBS 129.87	AF210676	N/A	AF358195	N/A	N/A	Schroers (2001)
***C.reniformis***	**CBS 695.86**	**OQ910685**	**OQ911044**	**OQ982714**	**OQ944697**	**OQ927753**	Zhao et al. (2023)
*C.rhinolophicola*	KUMC 21-0438	ON426841	N/A	OR025936	N/A	N/A	Liu et al. (2023)
***C.rhinolophicola***	**HKAS122257**	**ON426840**	N/A	**OR025937**	N/A	N/A	Liu et al. (2023)
***C.rhizophaga***	**CBS 202.37**	**AF358225**	**MH867396**	**AF358156**	N/A	N/A	Schroers (2001)
*C.rogersoniana*	CBS 582.89	AF210691	N/A	AF358189	N/A	N/A	Schroers (2001)
*C.rosea*	CBS 1221.71	DQ674381	OQ911077	OQ982747	OQ944730	OQ927786	Zhao et al. (2023)
*C.samuelsii*	CBS 699.97	OQ910812	N/A	AF358190	N/A	N/A	Zhao et al. (2023)
*C.setosa*	CBS 834.91	AF210670	N/A	AF358211	N/A	N/A	Schroers (2001)
*C.solani*	CBS 101924	AF358232	OQ911196	AF358180	OQ944847	OQ927902	Schroers (2001)
***C.spinulosa***	**MFLU 17-0131**	**ON230049**	N/A	**ON238009**	N/A	N/A	Perera et al. (2023)
***C.sporodochialis***	**CBS 101921**	**AF210685**	N/A	**AF358149**	N/A	N/A	Schroers (2001)
***C.squamuligera***	**PAD S00020**	**MT554908**	N/A	N/A	N/A	N/A	Forin et al. (2020)
*C.squamuligera*	PAD S00021	MT554909	N/A	N/A	N/A	N/A	Forin et al. (2020)
***C.subquaternata***	**CBS 100003**	**MT537603**	N/A	N/A	N/A	N/A	Forin et al. (2020)
***C.vacuolata***	**CBS 191.93**	**OQ910868**	**OQ911227**	N/A	**OQ944876**	**OQ927931**	Zhao et al. (2023)
***C.venezuelae***	**CBS 107.87**	**OQ910869**	**OQ911228**	**OQ982884**	**OQ944877**	**OQ927932**	Zhao et al. (2023)
***C.vesiculosa***	**HMAS 183151**	**NR_119828**	**HM050302**	N/A	N/A	N/A	Luo and Zhuang (2010)
***C.viticola***	**CAA 944**	**MK156282**	**N/A**	**MK156290**	**MK156286**	N/A	Torcato et al. (2020)
***C.viticola***	**MFLU 18-2770**	**MT215573**	**MT396164**	**N/A**	**MT212204**	N/A	Perera et al. (2020)
***C.wenpingii***	**HMAS 172156**	**NR_119651**	**MH874867**	N/A	N/A	N/A	Luo and Zhuang (2007)
***C.yadongensis***	**HKAS 133183**	**PQ522508**	**PQ634395**	**PQ650463**	**PQ650481**	**PQ538524**	present study
*C.yadongensis*	HKAS 133184	PQ522509	PQ634396	PQ650464	PQ650482	PQ538525	present study
***C.zelandiaenovae***	**CBS 100979**	**AF358229**	**OQ911231**	N/A	**OQ944880**	**OQ927935**	Schroers (2001)
*C.zelandiaenovae*	CBS 232.80	AF210684	N/A	AF358185	N/A	N/A	Schroers (2001)
*Mycocitruscoccicola*	HD 2016	KU720552	KU720545	N/A	N/A	N/A	Dao et al. (2016)
*M.coxeniae*	BRIP 49559a	OQ629341	N/A	N/A	N/A	N/A	Zhao et al. (2023)
***Sesquicilliumaquaticum***	**HKAS 125804**	**OP876724**	**OP875077**	N/A	N/A	N/A	Bao et al. (2023)
*S.buxi*	CBS 696.93	AF210667	KM231721	AF358215	KM231977	KM232416	Schroers (2001)
*S.candelabrum*	CBS 504.67	AF210668	N/A	N/A	N/A	N/A	Schroers (2001)
***S.candelabrum***	**YFCC 896**	**MW199067**	N/A	**MW201674**	N/A	N/A	Wang et al. (2023)
***S.essexcoheniae***	**BRIP 75170a**	**OQ629342**	N/A	N/A	**OQ944511**	**OQ914830**	Zhao et al. (2023)
***S.phyllophila***	**CBS 921.97**	**NR_137531**	N/A	N/A	N/A	N/A	Schroers (2001)
*S.rossmaniae*	CBS 210.93	AF358227	N/A	AF358213	N/A	N/A	Vu et al. (2019)
***S.saulense***	**BRFM 2782**	**MK635054**	N/A	N/A	N/A	N/A	Lechat et al. (2020)
***S.sesquicillii***	**CBS 180.88**	**AF210666**	**NG_228796**	**AF358214**	**OQ944535**	N/A	Schroers (2001)
***S.shanghaiense***	**HMAS 351878**	**OL897002**	**OL897044**	N/A	N/A	N/A	Zhang et al. (2023)
*S.shanghaiense*	GZUIFR 21.916	OL897003	OL897045	N/A	N/A	N/A	Zhang et al. (2023)
***Fusariumacutatum***	**CBS 402.97**	**NR_111142**	N/A	**MT011051**	N/A	N/A	Luo and Zhuang (2007)
*Nectriacinnabarina*	CBS 279.48	AF163025	HM484754	HM484802	HM484649	N/A	Hirooka et al. (2011)

The newly generated sequences are in red. The type strains are indicated in bold. The synonymizing are indicated in green. N/A denotes the unavailable data in GenBank.

A rapid phylogenetic analysis was performed utilizing OFPT (Zeng et al. 2023) according to its standard protocol. The final phylogenetic analyses were carried out on the CIPRES Science Gateway platform (https://www.phylo.org), employing RAxML-HPC v.8 on XSEDE (8.2.12) for maximum likelihood (ML) estimation and MrBayes on XSEDE (3.2.7a) for Bayesian inference (BI). Phylogenetic results were represented by ML bootstrap values (MLB) equal to or greater than 70% and a posterior probability in Bayesian statistics (BYPP) equal to or exceeding 0.90. These values were displayed above each node in all resulting trees. For visualization purposes, the resulting phylograms were displayed using the FigTree v1.4.0 program. The final reorganization was accomplished using Adobe Illustrator 2020.

## ﻿Results

### ﻿Phylogenetic analyses

**Figure 1.**
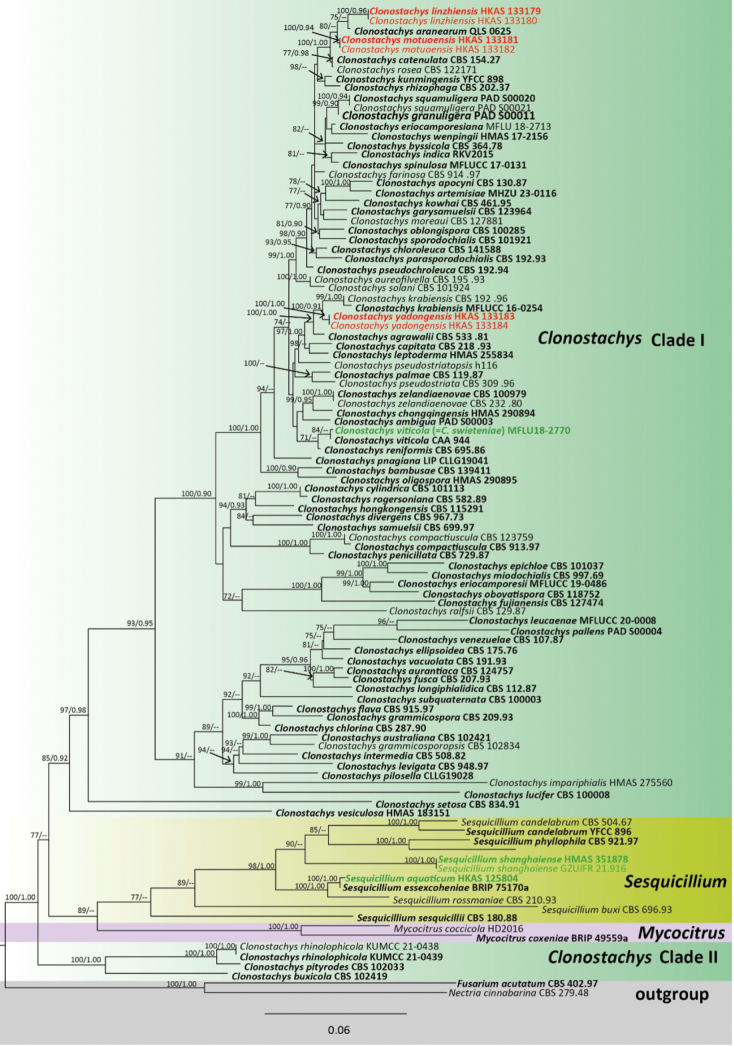
Phylogenetic tree generated from maximum likelihood analysis based on a combined 28S*tef*1, *rpb*2, ITS and *tub*2 sequence dataset. Bootstrap support values for ML equal to or greater than 70% and PP equal to greater than 0.90 are indicated at the nodes as MLB/BYPP. The ex-type strains are in bold, while the new isolates are in red, and the synonymizing taxa are indicated in green.

The combined 28S, *tef*1, *rpb*2, ITS, and *tub*2 dataset comprised 104 taxa. *Fusariumacutatum* (CBS 402.97) and *Nectriacinnabarina* (CBS 279.48) were selected as outgroup taxa (Prasher and Chauhan 2017; Lechat et al. 2020). The dataset consisted of 3146 total characters, including gaps (28S: 1–784 bp; *tef*1: 785–1596; *rpb*2: 1597–2349; ITS: 2350–2826; *tub*2: 2827–3828). The matrix had 1079 distinct alignment patterns, with 41.89% of undetermined characters or gaps. Estimated base frequencies were as follows: A = 0.229764, C = 0.268281, G = 0.268313, T = 0.233642; substitution rates: AC = 1.37920, AG = 4.09491, AT = 1.37920, CG = 0.794178, CT = 8.784537, GT = 1.00000; gamma distribution shape parameter α = 0.494958. The best-scoring RAxML tree with a final likelihood value of -23046.167770 is presented in (Fig. 1). Our specimens *Clonostachyslinzhiensis* (HKAS 133179 & HKAS 133180) and *C.motuoensis* (HKAS 133181 & HKAS 133182) formed distinct monophyletic clades with *C.aranearum* with support value of (75% ML) and (85% ML), indicating they are closely related. The two specimens HKAS 133183 and HKAS 133184 formed a sister clade to *C.krabiensis* with high support (100 ML/0.91 PP).

### ﻿Taxonomy

#### ﻿*Clonostachys*

##### ﻿ Clonostachys

Taxon classificationFungiHypocrealesBionectriaceae

Corda, Pracht-Fl. Eur. Schimmelbild: 31 (1839)

FBDBE180-C198-50DB-A046-6009B883BB13

Index Fungorum: IF7701

Facesoffungi Number: FoF02102

###### Classification.

Bionectriaceae, Hypocreales, Sordariomycetes.

###### Morphological characteristics.

**Sexual morph**: ***Ascomata*** perithecial. ***Perithecia*** superficial, solitary to gregarious, subglobose to globose, papillate or non-papillate, no colour change in 3% KOH or 100% LA. ***Asci*** clavate to subcylindrical, 6–8-spored. ***Ascospores*** ellipsoidal to oblong ellipsoidal, uniseptate, hyaline, smooth-walled, uniseriate or irregular biseriate. **Asexual morph**: Hyphomycetous. ***Conidiophores*** dimorphic or monomorphic, sporodochial, synnematous, hyaline, brown or blackish brown. ***Phialides*** phialidic, cylindrical to flask-shaped. ***Conidia*** aseptate, hyaline, smooth, ovoid to ellipsoid.

###### Type species.

*Clonostachysaraucaria* Corda, Pracht-Fl. Eur. Schimmelbild.: 31 (1839)

###### Notes.

*Clonostachys* is the second largest genus in Bionectriaceae, with 130 epithets (Index Fungorum 2025). Several members of *Clonostachys* are ecologically and economically important (Abeywickrama et al. 2023). Some *Clonostachys* spp. are destructive, including parasitic in myxomycetes, nematodes, ticks, molluscs, and leafhoppers (Schroers 2001; Toledo et al. 2006; Perera et al. 2023). *Clonostachysrosea* and *C.catenulata* are reported as destructive to ascomycetes and basidiomycetes (Schroers 2001; Chatterton et al. 2008) and *C.chuyangsinensis* and *C.aranearum* have been reported as spider-pathogenic fungi (Wan et al. 2016; Wang et al. 2023).

*Clonostachysrosea* has been studied as a potential biological control agent for various plant diseases and pests such as strawberry gray mold (Cota et al. 2008), *Fusarium* head blight of wheat (Xue et al. 2008), and *Pythiumtracheiphilum* in Chinese cabbage (Møller et al. 2003). Several closely related species to *Clonostachysrosea*, such as *C.byssicola*, *C.chloroleuca*, *C.rhizophaga*, and *C.solani* also possess biocontrol properties (Mendoza García et al. 2003; Krauss et al. 2013; Sun et al. 2017; Broberg et al. 2021).

##### ﻿ Clonostachys linzhiensis

Taxon classificationFungiHypocrealesBionectriaceae

S.C. He, K.D. Hyde & Q. Zhao sp. nov.

1CE081EA-EA63-5FC6-B3C9-947F82B9A3F3

Index Fungorum: IF902917

Facesoffungi Number: FoF16789

Fig. 2

###### Etymology.

The species epithet is derived from Linzhi City, where the holotype was collected.

###### Typification.

China • Xizang Autonomous Region, Linzhi City, Motuo County (29°11'N, 95°8'E, 1561 m), on the lower part of the leaves of *Houttuyniacordata*, July 27, 2022, collected by Hong-De Yang, YHD691 (**holotype**: KUN-HKAS 133179); ex-type living culture: KUNCC24-18528). GenBank: ITS: PQ522504, 28S: PQ634391, *tef*1: PQ650477, *tub*2: PQ650459.

###### Description.

**Sexual morph**: Not observed. **Asexual morph**: Hyphomycetous. ***Colonies*** on the WA, raised, medium sparse, rough, white at apex. ***Conidiophores*** mononematous, erect, simple, verticillium-like, straight or flexuous, branched, smooth-walled, thin-walled, septate, hyaline, produce globose cells at the apex, terminal branches developing into phialides, 110–232 × 2.5–3.9 μm (x̄ = 170 × 3.2 μm, n = 20). ***Phialides*** polytretic, terminal on branches, phialides cylindrical but slightly tapering towards the tips, aseptate, hyaline, smooth, thin-walled, terminal developing into conidia, 15.3–23.8 × 1.5–3.3 μm (x̄ = 19.8 × 2.2 μm, n = 20). ***Conidia*** amerospores, solitary, acrogenous, simple, doliiform to ellipsoidal, smooth, thin-walled, aseptate, hyaline, 3.9–5.7 × 2.2–3.2 μm (x̄ = 4.7 × 2.6 μm, n = 30).

**Figure 2.**
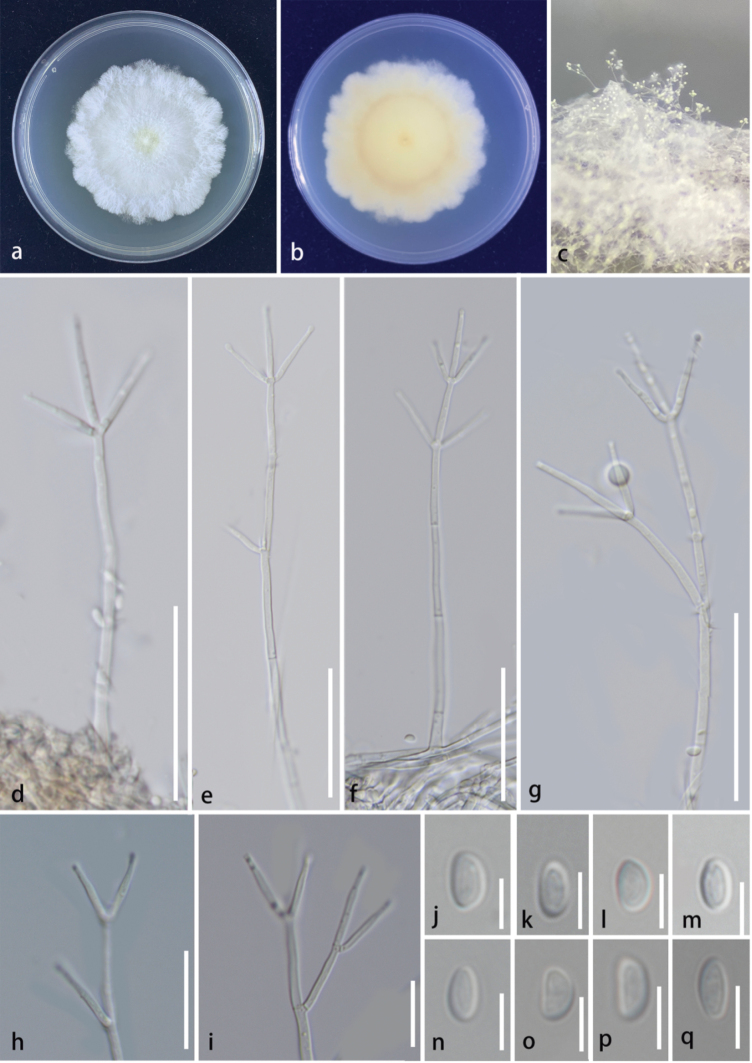
*Clonostachyslinzhiensis* (HKAS 133179, Holotype) **a, b** culture on PDA (**a** above **b** below) **c** colonies on WA**d–g** conidiophores **h, i** phialides **j–q** conidia. Scale bars: 50 μm (**d–g**); 50 μm (**h, i**); 5 μm (**j–q**).

###### Culture characteristics.

Colonies on PDA reaching 5.0–5.5 cm after 20 days of incubation at 25 °C, white above, pale yellow reverse, medium spare, concave in the center, convex around, hairy, lobate, velvety, ciliate, not pigment produced,

###### Habitat.

Leaves of *Houttuyniacordata*.

###### Additional material examined.

China • Xizang Autonomous Region, Linzhi City, Motuo County (29°11'N, 95°8'E, 1561 m), on the lower part of the leaves of *Houttuyniacordata*, July 27, 2022, collected by Hong-De Yang, HSC983 (**isotype**: KUN-HKAS 133180); ex-isotype living culture: KUNCC24-18529). GenBank: ITS: PQ522505, 28S: PQ634392, *tef*1: PQ650478, *tub*2: PQ650460.

###### Notes.

In the phylogenetic analysis, *Clonostachyslinzhiensis* shared a close phylogenetic relationship with *C.aranearum* and *C.motuoensis* (Fig. 1). *Clonostachyslinzhiensis* shares similar morphology to *C.aranearum* and *C.motuoensis* in having mononematous, erect, verticillium-like conidiophores that are straight or flexuous, smooth-walled, hyaline, phialides are polytretic, terminal, flask-shaped, aseptate, hyaline, smooth and the conidia are amerospores, acrogenous, ellipsoidal, aseptate, hyaline (Wan et al. 2016). However, *Clonostachyslinzhiensis* (HKAS 133179 and HKAS 133180) has larger conidiophores (L/W ratio: 53 vs 12 and L/W ratio: 53 vs 35) and longer phialides (L/W ratio: 9 vs 6.7 and L/W ratio: 9 vs 4.7) in comparison to *C.aranearum* and *C.motuoensis*. Furthermore, the ITS and *tub*2 sequence of *Clonostachyslinzhiensis* differs from *C.aranearum* which revealed 13/510 (2.5%) and 7/291 (2.4%) base pair differences, respectively. Based on the differences in morphology (larger conidiophores and longer phialides) and phylogeny, along with the guidelines of Maharachchimbukura et al. (2021), we identify our specimen as a new species, *C.linzhiensis*.

##### ﻿ Clonostachys motuoensis

Taxon classificationFungiHypocrealesBionectriaceae

S.C. He, K.D. Hyde & Q. Zhao sp. nov.

B6037BCA-496F-5A18-AAE3-6BE6055D2D80

Index Fungorum: IF902918

Facesoffungi Number: FoF16790

Fig. 3

###### Etymology.

The species epithet is derived from the location “Motuo County”, from where the holotype was collected.

###### Typification.

China • Xizang Autonomous Region, Linzhi City, Motuo County (29°11'N, 95°8'E, 1561 m), on the lower part of the leaves of *Houttuyniacordata*, July 27, 2022, collected by Hong-De Yang, YHD669-1 (holotype: KUN-HKAS HKAS 133181); ex-type living culture: KUNCC24-18530). GenBank:ITS: PQ522506, 28S: PQ634393, *tef*1: PQ650479, *tub*2: PQ650461.

###### Description.

**Sexual morph**: Not observed. **Asexual morph**: Hyphomycetous. ***Colonies*** on the WA, solitary or gregarious, white to pale yellow, raised, dense, rough. ***Conidiophores*** mononematous, penicillate, straight or flexuous, branched at the apex, smooth, thin-walled, septate, hyaline, conidiophores produce globose cells at the apex, from globose to elongated or continue to differentiate, terminal branches developing into phialides, 94–146 × 2.5–4.7 μm (x̄ = 125 × 3.5 μm, n = 20). ***Phialides*** monophialidic, terminal, flask-shaped, aseptate, hyaline, smooth, thin-walled, terminal developing into conidia, 9.1–18.7 × 2.3–3.5 μm (x̄ = 13.2 × 2.8 μm, n = 20). ***Conidia*** amerospores, solitary, acrogenous, simple, ellipsoidal to oblong with obtuse ends, smooth, thin-walled, aseptate, hyaline, minutely guttulate, 3.9–5.6 × 2.5–3.3 μm (x̄ = 4.6 × 2.9 μm, n = 30).

**Figure 3.**
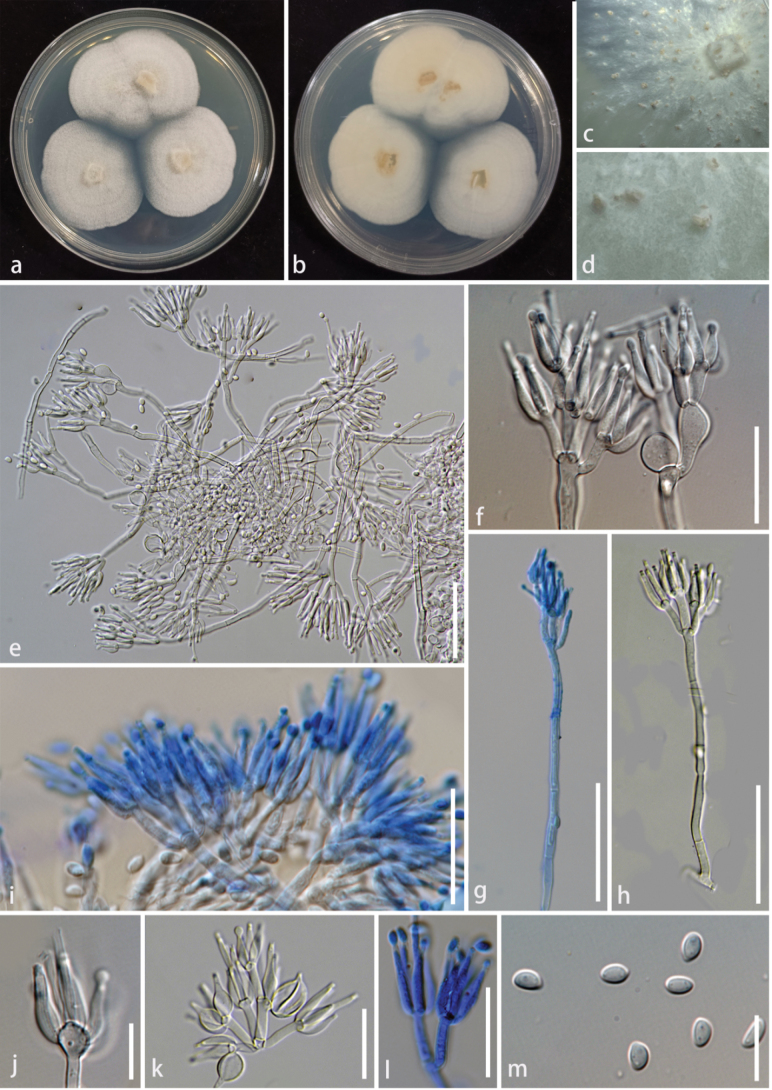
*Clonostachysmotuoensis* (HKAS 133181, Holotype) **a, b** culture on PDA (**a** above **b** below) **c, d** colonies on WA**e–h** conidiophores and conidiophores apex **i–l** phialides **m** conidia. Scale bars: 50 μm (**e, g, h**); 25 μm (**f, i, k, l**); 10 μm (**j, m**).

###### Culture characteristics.

Colonies on PDA reaching 3.5–4 cm after 20 days of incubation at 25 °C, white both above and reverse, medium spare, raised, smooth, fimbriate, velvety, ciliate, not pigment produced.

###### Habitat.

Leaves of *Houttuyniacordata*.

###### Additional material examined.

China • Xizang Autonomous Region, Linzhi City, Motuo County (29°11'N, 95°8'E, 1561 m), on the lower part of the leaves of *Houttuyniacordata*, July 27, 2022, collected by Hong-De Yang, HSC986 (isotype: KUN-HKAS 133182); ex-isotype living culture: KUNCC24-18531). GenBank: ITS: PQ522507, 28S: PQ634394, *tef*1: PQ650480, *tub*2: PQ650462.

###### Notes.

In the phylogenetic analysis, *Clonostachysmotuoensis* clustered sister to *C.linzhiensis* and *C.aranearum* (Fig. 1). Morphologically, our specimen (HKAS 133181 and HKAS 133182) has larger conidiophores (L/W ratio: 35 vs 12) and longer phialides (L/W ratio: 4.7 vs 6.7) in comparison to *C.aranearum*. *Clonostachysmotuoensis* differs from *C.aranearum* by 6/544 (1%) ITS and 4/294 (1.3%) *tub2* differences in the nucleotides. It is worth noting that *C.aranearum* is parasitic on spiders, while *C.motuoensis* is endophytic on *Houttuyniacordata* leaves. In addition, *C.aranearum* was collected from Qian Ling Shan Park, Guiyang City, Guizhou Province, China, with an altitude of 1100–1369 m, belonging to a plateau subtropical climate (Wan et al. 2016). *Clonostachysmotuoensis* was collected from Motuo County, Linzhi City, Xizang Autonomous Region, China, with an altitude of 1561 m, belonging to a tropical rainforest climate. Based on these distinctions and following the guidelines of Maharachchimbukura et al. (2021), we identified our specimen as a new species, *C.motuoensis*.

##### ﻿ Clonostachys yadongensis

Taxon classificationFungiHypocrealesBionectriaceae

S.C. He, K.D. Hyde & Q. Zhao sp. nov.

47BAC712-6287-5A66-978F-A4B9754C9ACD

Index Fungorum: IF902919

Facesoffungi Number: FoF16791

Fig. 4

###### Etymology.

The species epithet is derived from Yadong County, where the holotype was collected.

###### Typification.

China • Xizang Autonomous Region, Linzhi City, Yadong County (27°48'N, 88°83'E, 3894 m), on the lower part of the leaves of *Ageratinaadenophora* leaves, July 24, 2023, collected by Shu-Cheng He, HSC1025 (holotype: KUN-HKAS 133183); ex-type living culture: KUNCC24-18532). GenBank:ITS: PQ522508, 28S: PQ634395, *tef*1: PQ650481, *tub*2: PQ650463, *rpb2*: PQ538524.

###### Description.

**Sexual morph**: Not observed. **Asexual morph**: Hyphomycetous. ***Colonies*** on the WA, solitary or gregarious, white to pale yellow, raised, medium sparse, rough. ***Conidiophores*** mononematous, penicillate, straight or flexuous, branched, smooth-walled, thin-walled, septate, hyaline, produce globose cells at the apex, terminal branches developing into phialides, 80–118 × 2.4–3.4 μm (x̄ = 97 × 2.8 μm, n = 20). ***Phialides*** polyblastic, terminal, flask-shaped, aseptate, hyaline, smooth, thin-walled, minutely guttulate, terminal developing into conidia, 9.6–15.6 × 1.7–2.3 μm (x̄ = 13.1 × 2 μm, n = 20). ***Conidia*** amerospores, solitary, acrogenous, simple, oval to ellipsoidal, smooth, thin-walled, aseptate, hyaline, minutely guttulate, 3.6–5.4 × 2.6–3.3 μm (x̄ = 4.5 × 2.9 μm, n = 30).

**Figure 4.**
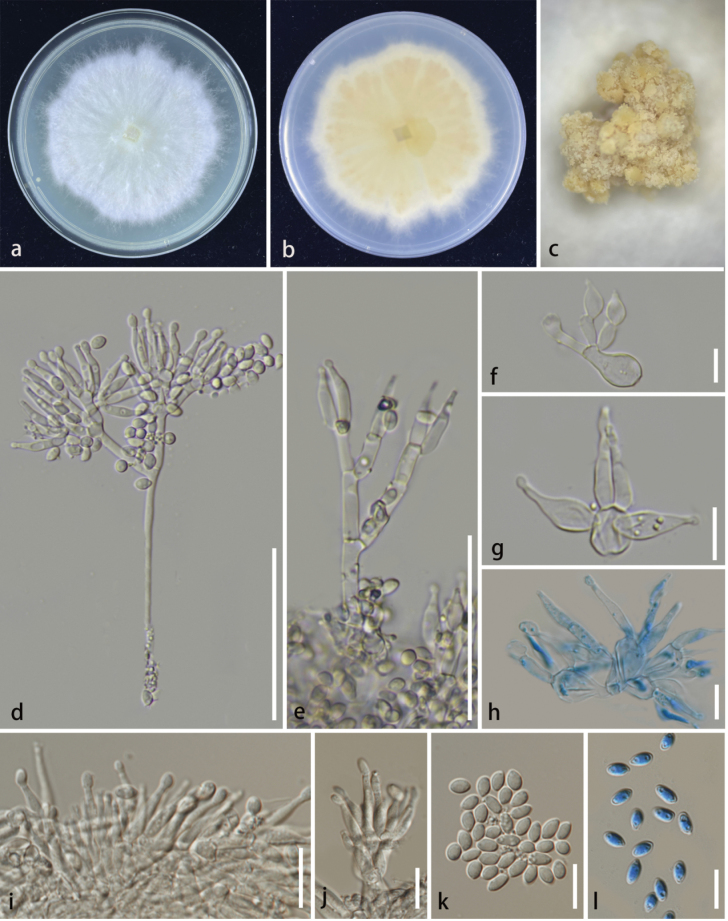
*Clonostachysyadongensis* (HKAS 133183, Holotype) **a, b** culture on PDA (**a** above **b** below); **c** colonies on WA**d–h** conidiophores **f–j** phialides **k, l** conidia. Scale bars: 50 μm (**d–f**); 20 μm (**g–l**).

###### Culture characteristics.

Colonies on PDA reaching 5.5–6 cm after 20 days of incubation at 25 °C, white above, pale yellow reverse, medium spare, raised, hairy, fimbriate, velvety, ciliate, not pigment produced.

###### Habitat.

Leaves of *Ageratinaadenophora*.

###### Additional material examined.

China • Xizang Autonomous Region, Linzhi City, Yadong County (27°48'N, 88°83'E, 3894 m), on the lower part of the leaves of *Ageratinaadenophora*, July 24, 2023, collected by Shu-Cheng He, HSC1025A (isotype: KUN-HKAS 133184; ex-isotype living culture: KUNCC24-18533). GenBank:ITS: PQ522509, 28S: PQ634391, *tef*1: PQ650482, *tub*2: PQ650464, *rpb2*: PQ538525.

###### Notes.

In the phylogenetic analysis, *Clonostachysyadongensis* clustered with *C.krabiensis* with 100% MLB and 0.91 BYPP support (Fig. 1). *Clonostachyskrabiensis* was introduced by Tibpromma et al. (2018) and is characterized by solitary, superficial, globose to subglobose, orange to brownish orange ascomata, 6–8-spored, cylindrical to clavate asci; fusoid to ellipsoidal, hyaline, with longitudinal striations, granulate ascospores. Its morphology fits well with the generic concept of *Clonostachys* sexual morph (Bao et al. 2023; Perera et al. 2023; Zhao et al. 2023). Our specimen (HKAS 133183) exhibited an asexual morph that is characterized by mononematous, penicillate, erect conidiophores; flask-shaped or cylindrical, aseptate, hyaline phialides; acrogenous, ellipsoidal or oblong with obtuse ends, hyaline conidia. The 28S and ITS sequences of *Clonostachysyadongensis* differ from that of *C.krabiensis* which showed base pair differences, 3/825 (0.35%), 11/513) and (2.1%) respectively. *Clonostachyskrabiensis* was reported in Papua New Guinea and Thailand as a saprobe on *Pandanus* sp. and wood litter, while *C.yadongensis* was reported in the Xizang Autonomous Region, China, mainly as an endophyte on *Ageratinaadenophora*. *Clonostachyskrabiensis* has been reported to have a sexual morph, but *C.yadongensis* has only been observed in its asexual morph. Based on base pair differences and following the guidelines of Maharachchimbukura et al. (2021), we identified our specimen as a new species, *Clonostachysyadongensis*.

##### ﻿ Clonostachys viticola

Taxon classificationFungiHypocrealesBionectriaceae

C. Torcato & A. Alves, Int. J. Syst. Evol. Microbiol, 6 (2020)

A51DC5C8-09AC-52B0-9255-010571E3BCF4

Index Fungorum: IF835021

Facesoffungi Number: FoF16792

###### Basionym.

*Clonostachysswieteniae* R.H. Perera, E.B.G. Jones & K.D. Hyde, *Mycosphere* 11(1): 2135 (2020)

###### Description and illustration.

Perera et al. 2020 and Torcato et al. 2020.

###### Notes.

In the multigene phylogenetic analyses, *Clonostachysviticola* with *C.swieteniae*, forms a monophyletic clade in *Clonostachys*. The taxa in this clade show low genetic differences. Thus, we recommend treating *C.viticola* and *C.swieteniae* as conspecific. *Clonostachysviticola* was established by Torcato et al. (2020) from the root of *Vitisvinifera* in a terrestrial habitat of Peru (Torcato et al. 2020) and *Clonostachysswieteniae* was established by Perera et al. (2020) from decaying fruits of *Swieteniamahagoni* in a terrestrial habitat of Thailand (Perera et al. 2020). Morphologically, *C.viticola* with *C.swieteniae* are highly similar, but there are minor differences in phialides (13.1 × 2.1 μm vs 11.4 × 2.6 μm), and conidia (5.6 × 2.9 μm vs 6 × 2.2 μm). Through base pair comparison, the ITS and *tef*1 sequence of *Clonostachysviticola* differs from that of *C.swieteniae* in 0/500 (0%) and 3/406 (0.7%), respectively. The results indicate that different environments have shaped the morphology (Bhunjun et al. 2022; Hyde et al. 2020b; Phukhamsakda et al. 2022). *Clonostachysviticola* was published prior to *C.swieteniae*. Therefore, we propose *C.swieteniae* as a synonym of *C.viticola*.

#### ﻿New combinations of *Sesquicillium*

##### ﻿ Sesquicillium

Taxon classificationFungiHypocrealesBionectriaceae

W. Gams, Acta bot. neerl. 17(6): 455 (1968)

1A0F5493-7137-5C5F-A4EF-7B21BBDF4DB4

Index Fungorum: IF9906

Facesoffungi Number: FoF16793

###### Classification.

Bionectriaceae, Hypocreales, Sordariomycetes

###### Morphological characteristics.

**Sexual morph**: Ascomycetous. ***Perithecia*** solitary, gregarious or loosely aggregated, globose to subglobose, 200–400 μm diam, pale yellow or pale to light orange, not papillate, Perithecial wall either consisting of two or one major wall regions. ***Asci*** clavate, 8-spored, with flat or rounded apex. ***Ascospores*** aseptate or 1-septate, hyaline, spinulose, warted, with short striae, ellipsoidal to fusiform. **Asexual morph.** Hyphomycetous. ***Conidiophores*** macronematous, mononematous, monomorphic or dimorphic, penicillate, verticillate; branches at apex. ***Phialides*** one or two successive intercalary phialides, terminal, terminal whorls consisting of narrowly flask-shaped, hyaline. ***Conidia*** obovoid, ellipsoid, or fusoid, slightly curved or straight, hyaline, aseptate, smooth-walled, thin-walled.

###### Type species.

*Sesquicilliumbuxi* (J.C. Schmidt ex Link) W. Gams, Acta bot. neerl. 17(6): 455 (1968)

###### Notes.

*Sesquicillium* was established by Gams (1968). Morphologically, *Sesquicillium* shares similar characteristics with *Clonostachys* in that the conidiophores are macronematous, monomorphic or dimorphic, penicillate, verticillate-like, branched, flask-shaped conidiogenous cells (Preedanon et al. 2023; Zhao et al. 2023). Zhao et al. (2023) revealed the close relationship between *Clonostachys* and *Sesquicillium* and reclassified eight species of *Clonostachys* to *Sesquicillium*. The difference between *Sesquicillium* and *Clonostachys* lies in the development of their conidiophores. In *Sesquicillium*, the conidiophore will form a lateral conidia process after bifurcation, leading to the production of conidia. In *Clonostachys*, the conidiophore will not form lateral conidia protrusions after bifurcation. It continues to differentiate into terminal phialides (Gams 1968; Schroers 2001). Based on the research of Chen et al. (2023), and Zhao et al. (2023), we used ITS, 28S, *tef*1, *tub*2, and *rpb*2 to reconstruct a phylogenetic tree to investigate the relationship of *Clonostachys* species. The results show that *Clonostachysaquatica* and *C.shanghaiensis* are far from *Clonostachys* and more closely related to *Sesquicillium*. Therefore, based on morphological and phylogenetic analysis, we propose *C.aquatica* and *C.shanghaiensis* are synonyms of *S.aquaticum* and *S.shanghaiense.*

##### ﻿ Sesquicillium aquaticum

Taxon classificationFungiHypocrealesBionectriaceae

(D.F. Bao, K.D. Hyde & Z.L. Luo) S.C. He, K.D. Hyde & Jayaward, [as ‘aquatica’] comb. nov.

4E2F58A7-F517-54B1-9252-8C1819871A51

Index Fungorum: IF903022

Facesoffungi Number: FoF16794

###### Basionym.

*Clonostachysaquatica* D.F. Bao, K.D. Hyde & Z.L. Luo, *Fungal Diversity*, (2023).

###### Holotype.

HKAS 125804.

###### Description and illustration.

See Bao et al. 2023.

###### Notes.

*Clonostachysaquatica* was established by Bao et al. (2023) based on ITS and *tub2* sequence data (holotype HKAS 125804). Through the study of Bao et al. (2023), *C.aquatica* clustered as a clade sister to *C.rossmaniae* with strong support (94% MLB, 98% MYPP). Following Bao et al. (2023), we added 28S, *tef*1 and *rpb*2 sequence data, and the results showed that *C.aquatica* clustered with *Sesquicilliumessexcoheniae* (100% MLB, 0.97 BYPP), forming a successive sister clade with *S.rossmaniae* (99% MLB,/1.00 BYPP) (Fig. 1). *Clonostachysaquatica* shows a closer relationship with *Sesquicillium* in phylogenetic analysis. Therefore, based on phylogenetic analysis, we propose *C.aquatica* as a synonym of *S.aquaticum*.

##### ﻿ Sesquicillium shanghaiense

Taxon classificationFungiHypocrealesBionectriaceae

(Zhi Yuan Zhang, Y.F. Han & Z.Q. Liang) S. C. He, K.D. Hyde & Jayaward, [as ‘shanghaiensis’] comb. nov.

65F6ECAE-541C-5621-A893-BF016B004401

Index Fungorum: IF903023

Facesoffungi Number: FoF16795

###### Basionym.

*Clonostachysshanghaiensis* Zhi Yuan Zhang, Y.F. Han & Z.Q. Liang, *MycoKeys* 98: 198 (2023).

###### Holotype.

HMAS 351878.

###### Description and illustration.

Zhang et al. (2023).

###### Notes.

*Clonostachysshanghaiensis* was established by Zhang et al. (2023), based on ITS and *tub2* sequence data (HMAS 351878). *Clonostachysshanghaiensis* clustered as a sister clade to *C.rossmaniae* (95% MLB, 0.99 BYPP) (Zhang et al. 2023). In this study, phylogenetic analysis showed that *Clonostachysshanghaiensis* formed a successive sister clade with *S.phyllophila*, *S.saulensis*, and *S.candelabrum* (Fig. 1). It is worth noting that *S.phyllophila*, *S.saulense*, and *S.candelabrum* were renamed by Zhao et al. (2023) as *C.phyllophila* (Schroers 2001), *C.saulensis* (Lechat et al. 2020), *C.candelabrum* (Schroers 2001) and *C.chuyangsinensis* (Wang et al. 2023) based on morphology and phylogenetic analysis. Therefore, based on phylogenetic analysis, we propose *C.shanghaiensis* as a synonym of *S.shanghaiense.*

## ﻿Discussion

Rossman et al. (2001) studied the asexual species in 15 genera of Bionectriaceae (Hypocreales) using 28S sequence data and showed that Bionectriaceae formed a monophyletic group. Recently, additional DNA gene sequences such as *acl*1, *tub*2, *rpb*1, and *tef*1 have been used to enhance the precision of phylogenetic trees within the *Clonostachys*/*Bionectria* species (Moreira et al. 2016). However, available sequence data for these four protein-encoding gene regions is lacking in GenBank (Moreira et al. 2016). Wang et al. (2023), stated that *tef*1 sequence data showed the highest resolution for distinguishing *Clonostachys* species (*tef*1>*tub*2>ITS) based on the investigation conducted for genetic divergence comparisons of *Clonostachys*. Zhao et al. (2023) investigated the generic delineation with broad taxon sampling with morphology and multi-gene (ITS, 28S, *tef*1, *tub*2, *rpb*2) phylogenetic analysis and found a close relationship to *Sesquicillium*. Further, *Sesquicillium* was resurrected to accommodate the former subgenera *Epiphloea* and *Uniparietina* (Zhao et al. 2023). We constructed a phylogenetic tree (Fig. 1) of *Clonostachys* based on five genes (28S, *tef*1, *rpb*2, ITS, and *tub*2) and show that *Clonostachys*/*Bionectria* form a similar topology with Perera et al. (2023). However, as with other studies, we did not achieve a well-supported clade, as some but not all subgenera are mono- or paraphyletic (Moreira et al. 2016; Bao et al. 2023; Perera et al. 2023; Wang et al. 2023; Zhao et al. 2023). Morphologically, the asexual morphs of *Clonostachys* exhibit similarities with those of *Sesquicillium* (Preedanon et al. 2023), *Penicillium* (Crous et al. 2023), *Verticillium* (Crous et al. 2022), *Gliocladium* (Rehner and Samuels 1994) acremonium-like (Preedanon et al. 2023). They typically feature macronematous, monomorphic penicillate, or dimorphic penicillate conidiophore. Based on recent studies by Bao et al. (2023), Wang et al. (2023), and Zhao et al. (2023), we have clarified the relationships within the *Clonostachys* and proposed that *C.aquatica*, *C.shanghaiensis*, and *C.swieteniae* be considered synonyms of *S.aquaticum*, *S.shanghaiense*, and *C.viticola*, respectively. *Clonostachysaquatica* and *C.shanghaiensis* were positioned in a distantly related clade (Clade II) to *Clonostachyssensu stricto.Mycocitrus* and *Sesquicillium*, were positioned between Clade I and II (Fig. 1). Thus, further studies are required for the phylogenetic resolution of *Clonostachys*.

*Clonostachys* is reported in various plant hosts: Apocynaceae, Arecaceae, Asteraceae, Boraginaceae, Buxaceae, Ericaceae, Fagaceae, Leguminosae, Melampsoraceae, Nelumbonaceae, Pandanaceae, Rosaceae, and Rutaceae (Wang et al. 2023; Jayawardena et al. 2025). Our study reported three new species from Eupatorieae (*C.yadongensis*) and Saururaceae (*C.linzhiensis* and *C.motuoensis*). *Clonostachys* species exhibit a saprobic or endophytic lifestyle, playing crucial roles in nutrient cycling and plant health (Zeng and Zhuang 2022). *Clonostachys* species are significant for their adaptability and potential as biological control agents against plant pathogens (Wang et al. 2023; Zhao et al. 2023).

## Supplementary Material

XML Treatment for Clonostachys

XML Treatment for Clonostachys linzhiensis

XML Treatment for Clonostachys motuoensis

XML Treatment for Clonostachys yadongensis

XML Treatment for Clonostachys viticola

XML Treatment for Sesquicillium

XML Treatment for Sesquicillium aquaticum

XML Treatment for Sesquicillium shanghaiense

## ﻿Additional information

### Author ORCIDs

Shucheng He https://orcid.org/0009-0008-7364-4727

Vinodhini Thiyagaraja https://orcid.org/0000-0002-8091-4579

Chitrabhanu S. Bhunjun https://orcid.org/0000-0001-8098-3390

Putarak Chomnunti https://orcid.org/0000-0003-2989-1735

Lakmali S. Dissanayake https://orcid.org/0000-0003-2933-3127

Ruvishika S. Jayawardena https://orcid.org/0000-0001-7702-4885

Yun Wei Zhao https://orcid.org/0009-0006-8211-5232

Fatimah Al-Otibi https://orcid.org/0000-0003-3629-5755

Qi Zhao https://orcid.org/0000-0001-8169-0573

Kevin D. Hyde https://orcid.org/0000-0002-2191-0762

### Data availability

All of the data that support the findings of this study are available in the main text.
